# Cortical Activation During Shoulder and Finger Movements in Healthy Adults: A Functional Near-Infrared Spectroscopy (fNIRS) Study

**DOI:** 10.3389/fnhum.2020.00260

**Published:** 2020-07-08

**Authors:** Chieh-Ling Yang, Shannon B. Lim, Sue Peters, Janice J. Eng

**Affiliations:** ^1^Department of Physical Therapy, Faculty of Medicine, University of British Columbia, Vancouver, BC, Canada; ^2^Rehabilitation Research Program, GF Strong Rehabilitation Centre, Vancouver, BC, Canada; ^3^Graduate Programs in Rehabilitation Sciences, Faculty of Medicine, University of British Columbia, Vancouver, BC, Canada

**Keywords:** functional near-infrared spectroscopy, brain, optical imaging, upper extremity, trial length

## Abstract

Characterization of cortical activation patterns during movement of the upper extremity in healthy adults is helpful in understanding recovery mechanisms following neurological disorders. This study explores cortical activation patterns associated with movements of the shoulder and fingers in healthy adults using functional near-infrared spectroscopy (fNIRS). Twelve healthy right-handed participants were recruited. Two motor tasks (shoulder abduction and finger extension) with two different trial lengths (10 s and 20 s) were performed in a sitting position at a rate of 0.5 Hz. The hemodynamic response, as indicated by oxy-hemoglobin (HbO) and deoxy-hemoglobin (HbR), over both hemispheres was acquired using a 54-channel fNIRS system. We found a generalized bilateral cortical activation during both motor tasks with greater activation in the contralateral compared to the ipsilateral primary motor cortex. Particularly in the more medial part of the contralateral hemisphere, significant higher activation was found during the shoulder compared to finger movements. Furthermore, cortical activation patterns are affected not only by motor tasks but also by trial lengths. HbO is more sensitive to detect cortical activation during finger movements in longer trials, while HbR is a better surrogate to capture active areas during shoulder movement in shorter trials. Based on these findings, reporting both HbO and HbR is strongly recommended for future fNIRS studies, and trial lengths should be taken into account when designing experiments and explaining results. Our findings demonstrating distinct cortical activation patterns associated with shoulder and finger movements in healthy adults provide a foundation for future research to study recovery mechanisms following neurological disorders.

## Background

Hand and arm motor tasks are commonly used to assess impaired motor function and to predict recovery in individuals with neurological disorders such as stroke. The ability to voluntarily extend the fingers and abduct the shoulder within 72 h after stroke onset predicts upper extremity functional recovery at 6 months (Nijland et al., 2010; Stinear et al., 2012, 2017). Moreover, people with stroke with good recovery typically show relatively normal task-related brain activation, whereas those with poor recovery tend to recruit additional brain regions when compared to healthy controls during a motor task (Ward et al., 2003). Therefore, characterization of brain activation patterns during the execution of such movements (i.e., shoulder abduction and finger extension) in healthy individuals can be useful to study recovery mechanisms following neurological disorders, develop new strategies for upper extremity rehabilitation, and better predict functional recovery.

Functional near-infrared spectroscopy (fNIRS) is a neuroimaging technique that has several advantages including being portable and non-invasive. It has a wide application in the field of rehabilitation (e.g., gait rehabilitation, cognitive assessment, brain-machine interface, a combined modality with electroencephalography, and influence of external stimulation assessment) and in different populations (e.g., stroke, mild cognitive impairment, Parkinson’s disease; Zafar and Hong, 2016; Khan M. J. et al., 2018; Khan R. A. et al., 2018; Yaqub et al., 2018; Bandeira et al., 2019; Curtin et al., 2019; Ghafoor et al., 2019; Li et al., 2019; Yang et al., 2019). Furthermore, as fNIRS is less sensitive to motion artifact compared with some neuroimaging modalities, real-time cortical activity during movement of proximal joints, such as the shoulder, can be undertaken where the usage of current traditional neuroimaging tools (e.g., fMRI) is limited (Strangman et al., 2006). fNIRS has been shown to be a promising neuroimaging technology that provides information regarding cortical activation through monitoring of blood oxygenation and blood volume in the cortex with a relatively good spatial (~1 cm) and temporal resolution (~0.1 s) compared to other neuroimaging techniques like EEG and fMRI, respectively (Herold et al., 2018). By measuring changes in near-infrared light attenuation at multiple wavelengths, fNIRS quantifies the concentration of oxy-hemoglobin (HbO) and deoxy-hemoglobin (HbR) reflecting neuronal activity *via* neurovascular coupling driven by adjustment of cerebral blood flow (Lecrux and Hamel, 2011; Boas et al., 2014). Neurovascular coupling is a mechanism of the relationship between local neural activity and subsequent adjustment of blood supply to the needs in energy and oxygen of activated neurons in the brain (Herold et al., 2018). As an increase in neural activity results in an increase in the oxygen metabolism, typical neuronal activity is assumed to be characterized by an increase in HbO and a simultaneous slight decrease in HbR due to the influx of cerebral blood flow to the active brain regions (Leff et al., 2011). Studies have demonstrated that fNIRS is reliable and valid to examine functional brain activity in various cortical regions during motor tasks in healthy and disease populations (Herold et al., 2017).

Structurally, based on the motor homunculus of a human brain, the area representing the musculature of the shoulder has been suggested to be located more medial compared to that of fingers in the primary motor cortex (Penfield and Boldrey, 1937). The musculature of distal joints, such as fingers, is controlled largely by the lateral corticospinal tract, where most of the corticospinal neurons originate in the primary motor cortex or the premotor and supplementary motor areas (Hall and Guyton, 2005). In contrast, the cortico-reticulospinal tract, which originates mainly from the premotor cortex, controls the proximal muscles of extremities, such as that of the shoulder (Mendoza and Foundas, 2009). Although movements of the body are principally controlled through the contralateral hemisphere, functional neuroimaging studies also show ipsilateral cortical activation with movements of the upper extremity (Ganguly et al., 2009; Diedrichsen et al., 2013). Previous functional neuroimaging studies have shown distinct cortical activation patterns during movements of different joints (Luft et al., 2002; Kapreli et al., 2006; LaPointe et al., 2009) but only one study using fNIRS examined the cortical activation differences between the cortical representation of the shoulder and hand (Yeo et al., 2013). Yeo et al. (2013) found greater activation of the prefrontal and premotor cortex with movements of the shoulder, while greater activation was found in the sensorimotor cortex for the hand. However, this study only examined the contralateral hemisphere and no studies to date have specifically investigated the cortical activation patterns of both hemispheres during shoulder and finger movements, which may be indicators of motor recovery following stroke. It is important to understand the functions of both hemispheres as stroke can result in compensatory activation from the non-lesioned hemisphere (Takeuchi and Izumi, 2012).

Trial lengths during upper extremity motor tasks in fNIRS studies have ranged from 10 s to 20 s in prior publications (Mehnert et al., 2013; Batula et al., 2017; Dravida et al., 2017; Lee et al., 2018; Sun et al., 2018; Wu et al., 2018). Long trial lengths result in participant burden especially in individuals with stroke who fatigue easily (Nadarajah and Goh, 2015). Shorter trials are preferred compared to longer trials if changes in brain activity can be captured by using shorter trials. One previous study has found that shorter trials may improve phase regularity of hemodynamic responses compared to longer trials during finger movements (Toronov et al., 2000). Another study has demonstrated that the amplitude of hemodynamic response attenuated over a long (i.e., 1-min) visual stimulating period (Obrig et al., 2002). However, no studies so far have examined the effect of trial length on cortical activation patterns measured by NIRS.

Thus, the primary objective of this study was to establish the cortical activation patterns associated with movements of shoulder and fingers in healthy. We hypothesize bilateral activation of sensorimotor areas with more activation in the contralateral hemisphere, activation in more lateral regions for finger movements, and activation of more medial regions for shoulder movements. A secondary objective of this study was to compare two trial lengths (e.g., 10 s vs. 20 s) that have been commonly used to examine brain activation during upper extremity motor tasks in fNIRS studies to examine the effect of trial length on the hemodynamic responses captured by NIRS. Based on the good temporal resolution of fNIRS, we hypothesize no difference in activation between shorter and longer trial lengths. Understanding the cortical activation patterns associated with neurological recovery, as well as the optimum trial length will be beneficial for clinical application and implementation of the fNIRS in the disease population.

## Materials and Methods

### Participants

Twelve healthy right-handed participants (mean age: 32.5 ± 9.4 years, range: 19–46, five males, seven females) with no history of neurological, physical, or psychiatric disease were recruited for this study. Hand dominance was determined based on the self-report of the writing hand. Written informed consent was obtained from all participants before participation. The study protocol was approved by the University of British Columbia Behavioural Research Ethics Board, study number H15-02782.

### fNIRS System and Probe Placement

**Figure 1:**
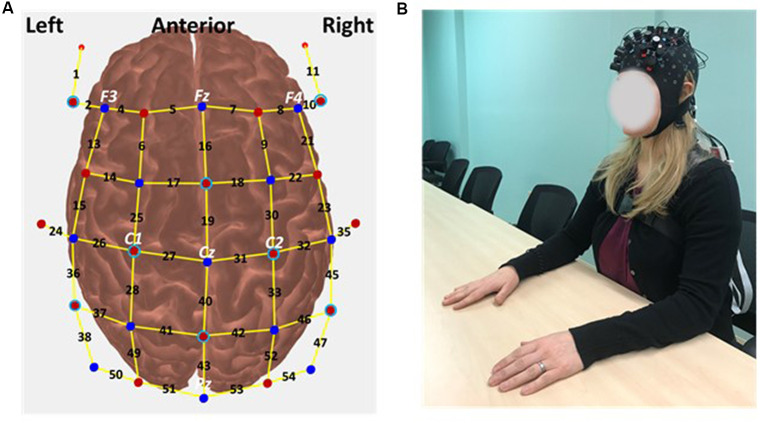
**(A)** The schematic view of the optodes. The red and blue solid circles indicate the position of sources and long separation detectors, respectively. The light blue open circles indicate the position of short separation detectors. The black enumeration corresponds to the channel number. The yellow lines indicate channels formed by source-long separation detector pairs. Brain regions of the channels are listed in Table 1. **(B)** Example of a participant with an easy cap with the optodes covering bilateral frontal and parietal lobes.

The continuous wave fNIRS system (NIRSport2, NIRx Medical Technologies) with LED optodes (wavelengths of 760 and 850 nm) was used to measure cortical activity at a sampling rate of 4.3597 Hz. The “fNIRS Optodes Location Decider (fOLD)” toolbox was used to guide the selection of optode positions to create a probe covering the sensorimotor and frontal cortices (Zimeo Morais et al., 2018). Using the fOLD toolbox, a list of the selected fNIRS channels and corresponding Brodmann’s areas were then created for ease of comparison with fMRI literature (Table 1). The probe contained 16 sources, 15 long separation detectors (approximately 3 cm distance from the source), and 8 short separation detectors (approximately 8 mm distance from each source; Brigadoi and Cooper, 2015; Yücel et al., 2015; Jahani et al., 2017; Nemani et al., 2018). This configuration resulted in a total of 54 channels in which 46 are long and 8 are short separation source-detector pairs (Figure 1A). The long separation channels are sensitive to measure hemoglobin changes in the cortex of the brain and superficial layers (i.e., the scalp and the skull). The short separation channels provide a measure of noise from the superficial layers only (Gagnon et al., 2011; Brigadoi and Cooper, 2015). The short separation channels were thus used to regress out the hemoglobin changes along with the superficial layers and isolate hemodynamic responses specific to activation in the brain (Gagnon et al., 2012; Brigadoi and Cooper, 2015; Yücel et al., 2015). Before the experiment, the head circumference, nasion-inion distance, and ear-to-ear distance (between preauricular points) of the participants were measured. An easy cap with the optodes inserted into the cap at defined International 10/20 system positions (Homan et al., 1987) was positioned on the participant’s head (Figure 1B).

**Table 1:** Brain regions of the channels and their corresponding *p*-values in oxyhemoglobin (HbO) and deoxyhemoglobin (HbR) for each condition.

Ch	Brodmann’s area	*P*-value for HbO				*P*-value for HbR			
		S-10 s	S-20 s	F-10 s	F-20 s	S-10 s	S-20 s	F-10 s	F-20 s
1	45-pars triangularis Broca’s area	0.5122	0.9095	0.3642	0.4823	0.9000	0.5654	0.5613	0.0020
2	45-pars triangularis Broca’s area	0.5491	0.3702	0.5398	0.1469	0.6935	0.9343	0.1627	0.0050
4	9-Dorsolateral prefrontal cortex	0.2510	0.8546	0.1545	0.1174	0.7702	0.7432	0.6703	0.0070
5	9-Dorsolateral prefrontal cortex	0.3076	0.7726	0.0452	0.1328	0.8119	0.0172	0.0730	0.0080
6	8-Includes Frontal eye fields	0.1222	0.7491	0.0074*	0.0673	0.0318	0.7849	0.4524	0.0090
7	9-Dorsolateral prefrontal cortex	0.1935	0.7090	0.1080	0.0686	0.4332	0.9274	0.1060	0.0130
8	9-Dorsolateral prefrontal cortex	0.4526	0.2902	0.1703	0.1163	0.0942	0.5210	0.1748	0.0190
9	8-Includes Frontal eye fields	0.1950	0.2262	0.1957	0.0031*	0.0055*	0.0010*	0.8865	0.0190
10	45-pars triangularis Broca’s area	0.2993	0.4572	0.3748	0.3025	0.0145*	0.0446	0.1319	0.0200
11	45-pars triangularis Broca’s area	0.9028	0.0774	0.4513	0.4550	0.0123*	0.0047*	0.1124	0.0240
13	9-Dorsolateral prefrontal cortex	0.8276	0.3309	0.7817	0.3555	0.4744	0.7934	0.2127	0.0330
14	6-Pre-Motor and Supplementary Motor Cortex	0.2749	0.7258	0.0875	0.1803	0.0811	0.2739	0.9622	0.0560
15	6-Pre-Motor and Supplementary Motor Cortex	0.0064*	0.0644	0.0003*	0.0032*	0.0132*	0.0490	0.0157	0.0630
16	8-Includes Frontal eye fields	0.0151	0.0181	0.0008*	<0.0001*	0.0057*	0.0395	0.6907	0.0760
17	6-Pre-Motor and Supplementary Motor Cortex	0.1036	0.7487	0.0154	0.0480	0.0007*	0.1406	0.8849	0.0830
18	6-Pre-Motor and Supplementary Motor Cortex	0.3851	0.5544	0.0203	0.0075*	0.0020*	0.0072	0.4214	0.0880
19	6-Pre-Motor and Supplementary Motor Cortex	0.6184	0.7745	0.6846	0.5411	0.0075*	0.2539	0.0382	0.0910
21	9-Dorsolateral prefrontal cortex	0.6368	0.3771	0.0146	0.0443	0.0127*	0.0491	0.9291	0.0940
22	6-Pre-Motor and Supplementary Motor Cortex	0.4994	0.1877	0.5090	0.2165	0.2673	0.8712	0.8308	0.1450
23	6-Pre-Motor and Supplementary Motor Cortex	0.3123	0.4886	0.6402	0.1738	0.2169	0.8862	0.6986	0.1540
24	3-Primary Somatosensory Cortex	0.0025*	0.0037*	0.0065*	<0.0001*	0.9657	0.7568	0.1259	0.1750
25	6-Pre-Motor and Supplementary Motor Cortex	0.0001*	0.0008*	0.0015*	0.0005*	0.0044*	0.0006*	0.0095	0.1760
26	4-Primary Motor Cortex	<0.0001*	0.0001*	0.0001*	<0.0001*	0.0028*	0.0004*	0.0030	0.2180
27	6-Pre-Motor and Supplementary Motor Cortex	0.0132	0.0266	0.0457	0.0131*	0.0087*	0.2604	0.0907	0.2550
28	4-Primary Motor Cortex	0.0009*	0.0004*^†^	0.0004*	0.0033*^†^	0.0076*	0.0075	0.0672	0.2800
30	6-Pre-Motor and Supplementary Motor Cortex	0.0540	0.3824	0.0585	0.1451	0.0538	0.3994	0.3793	0.2990
31	6-Pre-Motor and Supplementary Motor Cortex	0.4390	0.3887	0.1568	0.0145*	0.0502	0.6548	0.6194	0.3310
32	4-Primary Motor Cortex	0.5081	0.0907	0.5295	0.1100	0.2652	0.9166	0.9445	0.3640
33	4-Primary Motor Cortex	0.8843	0.7289	0.8239	0.0344	0.0111*	0.0950	0.4363	0.3900
35	2-Primary Somatosensory Cortex	0.0314	0.3336	0.2114	0.2503	0.0579	0.0636	0.1792	0.4580
36	40-Supramarginal gyrus part of Wernicke’s area	0.0008*	0.0022*	0.0011*	0.0024*	0.0032*	0.0041*	0.0059	0.4770
37	40-Supramarginal gyrus part of Wernicke’s area	0.0022*	0.0073*	0.0030*	0.0003*	0.0010*^†^	0.0282	0.1063^†^	0.4810
38	39-Angular gyrus, part of Wernicke’s area	0.0032*	0.0023*	0.0167	0.0405	0.0140*	0.2993	0.1602	0.4990
40	4-Primary Motor Cortex	0.0996	0.2605	0.1264	0.0515	0.4462	0.2236	0.7608	0.7150
41	5-Somatosensory Association Cortex	0.0310	0.0853	0.0236	0.0307	0.0074*	0.3292	0.6274	0.7800
42	5-Somatosensory Association Cortex	0.2103	0.1873	0.1033	0.0361	0.0051*	0.1466	0.8848	0.8710
43	7-Somatosensory Association Cortex	0.0286	0.8536	0.0281	0.0016*	0.0025*	0.0695	0.5625	0.9150
45	40-Supramarginal gyrus part of Wernicke’s area	0.2031	0.6081	0.0749	0.0468	0.1152	0.1263	0.4864	0.9820
46	40-Supramarginal gyrus part of Wernicke’s area	0.5140	0.7467	0.0520	0.0025*	0.0471	0.0860	0.8436	0.9950
47	39-Angular gyrus, part of Wernicke’s area	0.4284	0.3046	0.4736	0.1442	0.0025*	0.3452	0.1100	0.0020
49	7-Somatosensory Association Cortex	0.0584	0.0860	0.0702	0.0381	0.3343	0.4485	0.5479	0.0060
50	7-Somatosensory Association Cortex	0.8062	0.5810	0.2943	0.3062	0.0017*	0.3966	0.0004*	0.0070
51	7-Somatosensory Association Cortex	0.1356	0.0826	0.1125	0.0194*	0.3375	0.4178	0.9528	0.0080
52	7-Somatosensory Association Cortex	0.3908	0.0617	0.0167	0.0008*	0.3326	0.1754	0.3246	0.0090
53	7-Somatosensory Association Cortex	0.1438	0.0791	0.0024*	0.0001*	0.1641	0.8827	0.8533	0.0130
54	7-Somatosensory Association Cortex	0.8660	0.2993	0.0236	0.0008*	0.1121	0.9733	0.0595	0.0190

*Significant difference between baseline and task-related hemodynamic responses. ^†^Significant differences between conditions. Absolute *p*-values are shown in the table. The significant levels*^†^ are corrected for multiple comparisons using the Benjamini–Hochberg method with a false discovery rate of 0.05. Ch, Channel; S, Shoulder abduction task; F, Finger extension task. Channel 3, 12, 20, 29, 34, 39, 44, and 48 are short separation channels therefore were not listed.

### Experimental Design

**Figure 2:**
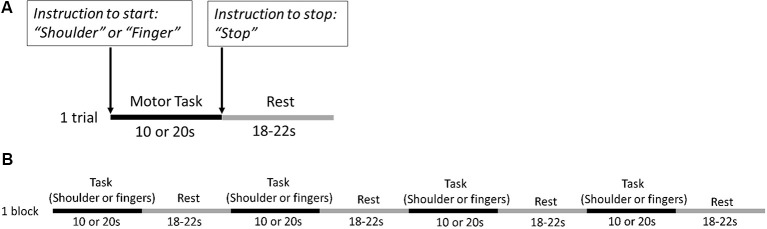
Example of **(A)** one trial and **(B)** one experimental block. The verbal instructions (i.e., Shoulder” or “Finger”) to start the motor tasks were given at the onset of each trial, and the instructions to stop the motor tasks (i.e., “Stop”) were given at the end of each trial. Four trials followed by inter-trial rest in each block with one trial for each condition (shoulder abduction for 10 s, shoulder abduction for 20 s, fingers extension for 10 s, and fingers extension for 20 s). The order of conditions was randomized in each block.

All participants were asked to sit on a chair with their back resting on the backrest. They were asked to perform four conditions (2 motor tasks × 2 task lengths): shoulder abduction or fingers extension for 10 or 20 s of trial lengths (i.e., shoulder-10 s, shoulder-20 s, finger-10 s, and finger-20 s). The experimental protocol included 20 blocks and each block included four trials (one trial for each condition), which resulted in 20 trials for each condition (Figure 2). The order of conditions was assigned randomly in each block using the PsychoPy program (Peirce et al., 2019) with the experimenter triggering the start to each block. The rest time between trials varied from 18 s to 22 s to minimize the physiological effects of breathing, heart rate, and Mayer waves (low-frequency arterial pressure oscillations) on the task hemodynamic responses (Leff et al., 2011).

Before the actual experiment, participants practiced both motor tasks (i.e., shoulder abduction and fingers extension), guided by the experimenter, to familiarize themselves with the tasks and instructions. During the shoulder abduction task, participants were instructed to abduct their right shoulder to approximately 45 degrees. During the finger extension task, participants were instructed to fully extend the second to fifth digits of their right hands. The motor tasks were performed under metronome guidance at a frequency of 0.5 Hz and the metronome was on during both motor tasks and rest periods to control for the effects of the auditory stimulus. The pace was visually inspected by the experimenter to ensure that participants adhered to the 0.5-Hz tempo. The verbal instructions to start the motor tasks were “Shoulder” and “Finger” for shoulder abduction and finger extension tasks, respectively, and the instruction to stop the motor tasks was “Stop.” Participants did not know which motor task was to be performed until the instructions for initiating the motor tasks (i.e., “Shoulder” or “Finger”) were given. These instructions were provided by the PsychoPy program. Participants were blinded to the length of each trial and were instructed not to count the number of their movement repetitions in each trial. During the testing, participants were instructed not to hold their breath to minimize the effects of blood pressure changes associated with breath-holding on hemodynamic responses. During the inter-trial rest periods, participants were asked to relax, refrain from moving, and not think about anything in particular. Participants were instructed to looked at a cross at eye level in front of them during motor tasks and rest periods to minimize head movements.

### Data Analysis

fNIRS data were processed by using open source software HOMER2 (Huppert et al., 2009) which is implemented in MATLAB (Mathworks, Natick, MA, USA). While some studies focused on channels that cover predetermined regions of interest (Mehnert et al., 2013; Yeo et al., 2013; Sun et al., 2018), we decided to utilize all channels to show cortical activation maps to serve a guide for the selection of regions of interest for future studies. First, the channels that are lower than 0.0005 V or higher than 1 V and had a signal to noise ratio less than 3 were excluded (Jahani et al., 2018). The raw fNIRS signal was converted to optical density by taking the logarithm of the signal and was then corrected for baseline shift and motion artifacts by the application of the Spline-SG method with parameter “*p*” equaled to 0.99 and a frame length of 10 s. Residual motion artifacts were then identified by any sudden increases in the optical density greater than 20 times the standard deviation and trials with residual motion artifacts were excluded from analysis (Jahani et al., 2018). A low-pass filter with a cut-off frequency of 0.5 Hz was applied to optical density signals to remove high-frequency noise. The optical signals were then converted to HbO and HbR concentration by applying the modified Beer–Lambert law with a partial pathlength factor of 6 (Boas et al., 2004). The hemodynamic response function was estimated by a general linear model approach which uses the ordinary least square method to estimate the weight of consecutive Gaussian functions with a standard deviation of 1 s and their means separated by 1 s (Gagnon et al., 2011; Jahani et al., 2017) over the regression time range of −2 s to 30 s. To correct for the drift, a third order polynomial fit was used to model the baseline drift. The short separation channel with the highest correlation with a given long separation channel was used as a regressor to cancel the physiological interferences from the long separation channel (Gagnon et al., 2011). For the behavioral data, the number of repetitions were counted for each trial by the experimenter to determine whether each condition was completed at a similar pace.

### Statistical Analysis

**Figure 3:**
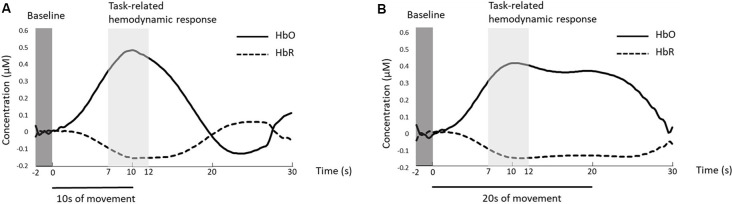
Example of a representative averaged hemodynamic response in channel 26 during **(A)** 10 s of shoulder abduction and **(B)** 20 s of shoulder abduction as shown by an increase in oxy-hemoglobin (HbO; solid line) and a slight decrease in deoxy-hemoglobin (HbR; dotted line). Baseline hemodynamic responses (dark gray box) were defined as the responses from 2 s before the task onset to the task onset. Task-related hemodynamic responses (light gray box) were defined as the responses from 7 s to 12 s after the task onset.

Changes in cortical activation were determined through a two-step method. For Step 1, paired *t*-tests for each channel were used to evaluate statistically significant differences in averaged hemodynamic responses between baseline (2 s immediately before the task onset) and task (7–12 s after the task onset) for each condition (Figure 3). Based on visual inspection, the time range of 7–12 s after the task onset (5 s time window) was chosen as the task-related hemodynamic responses for statistical analysis as participants reached their peaks in this time range. For Step 2, if a channel showed statistical differences from baseline under any condition, the channel was then evaluated for differences between conditions. Averaged task-related hemodynamic responses (7–12 s post-onset) during each condition as measured by HbO and HbR were compared using paired *t*-tests between: (1) shoulder abduction vs. fingers extension tasks with the same trial lengths (i.e., shoulder-10 vs. finger-10 and shoulder-20 vs. finger-20) to quantify the effect of motor tasks (primary objective); and (2) 10 s vs. 20 s trial lengths under the same motor task (i.e., shoulder-10 vs. shoulder-20 and finger-10 vs. finger-20) to understand the effects of trial lengths (secondary objective). To further understand the level of activation in major brain regions associated with upper extremity movements between hemispheres, we conducted *post hoc* analyses for the primary objective to compare the averaged task-related hemodynamic responses (7–12 s post-onset) between areas in the ipsilateral primary motor cortex and corresponding channels in the contralateral side (channels in ipsilateral vs. contralateral primary motor cortex: channel 26 vs. channel 32; channel 28 vs. channel 33 as shown in Table 1). Benjamini–Hochberg’s method with a false discovery rate of 0.05 was applied to correct for the multiple comparisons in both steps (Benjamini and Hochberg, 1995; Singh and Dan, 2006). For behavioral data, paired *t*-tests were used to examine statistically significant differences in the number of repetitions between conditions with the same trial lengths (i.e., shoulder-10 vs. finger-10, shoulder-20 vs. finger-20).

## Results

### Comparisons Between Baseline vs. Task-Related Hemodynamic Responses (Step 1)

**Figure 4:**
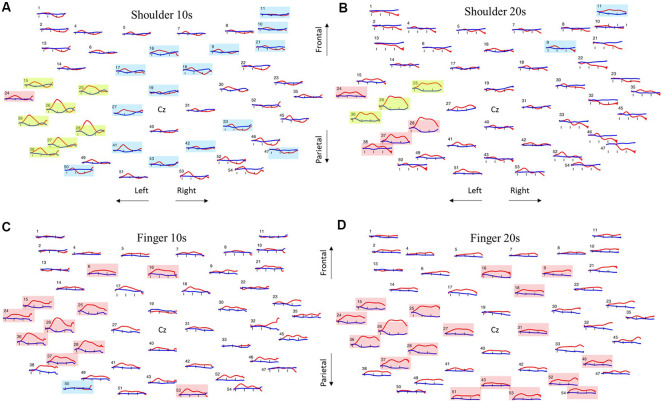
Group average results for all channels all four conditions with red traces indicating HbO and blue traces indicating HbR. **(A)** Shoulder abduction for 10 s. **(B)** Shoulder abduction for 20 s. **(C)** Finger extension for 10 s. **(D)** Finger extension for 20 s. Red boxes indicate a significant difference between baseline and task in HbO. Blue boxes indicate a significant difference between baseline and task in HbR. Green boxes indicate a significant difference between baseline and task in both HbO and HbR.

For all conditions (shoulder-10 s, shoulder-20 s, finger-10 s, and finger-20 s), common brain regions that showed a significant increase in HbO were contralateral premotor, contralateral supplementary motor cortex, contralateral primary somatosensory cortex, contralateral primary motor cortex, as well as contralateral supramarginal gyrus (Table 1 and Figure 4). Greater number of additional active areas were found for the finger compared to shoulder conditions; moreover, a greater number of additional active areas were found for the finger-20 s compared to finger-10 s (shoulder-10 s: eight channels, shoulder-20 s: seven channels, finger-10 s: 10 channels, finger-20 s: 18 channels out of 46 channels). For the shoulder-10 s and shoulder-20 s conditions (Figures 4A,B), an additional significant increase in HbO was found only in the contralateral angular gyrus. For the finger-10 s condition (Figure 4C), a significant increase in HbO was found in contralateral frontal eye fields and ipsilateral somatosensory association cortex. For the finger-20 s condition (Figure 4D), the ipsilateral premotor cortex, ipsilateral supplementary motor cortex, ipsilateral frontal eye fields, and bilateral somatosensory association cortex demonstrated significant increases in HbO.

For the shoulder-10 s and shoulder-20 s conditions, common brain regions that showed a significant decrease in HbR were ipsilateral frontal eye fields, pars triangularis Broca’s area, contralateral premotor, contralateral supplementary motor cortex, contralateral primary motor cortex, and contralateral supramarginal gyrus (Table 1, Figures 4A,B). Greater number of additional active areas were found for the shoulder compared to finger conditions; moreover, a greater number of additional active areas were found for the shoulder-10 s compared to shoulder-20 s (shoulder-10 s: 22 channels, shoulder-20 s: five channels, finger-10 s: one channel, finger-20 s: none out of 46 channels). For the shoulder-10 s (Figure 4A), additional areas demonstrating a significant decrease in HbR were ipsilateral premotor cortex, ipsilateral supplementary motor cortex, ipsilateral dorsolateral prefrontal cortex, ipsilateral primary motor cortex, contralateral angular gyrus, and bilateral somatosensory association cortex. During the finger-10 s condition (Figure 4C), only one channel in the contralateral somatosensory association cortex had a significantly greater increase in HbR during the task compared to the baseline (Table 1). No significant change in HbR was found during the finger-20 s condition (Figure 4D).

### Comparisons of Task-Related Hemodynamic Responses Between Different Motor Tasks and Trial Lengths (Step 2)

**Figure 5:**
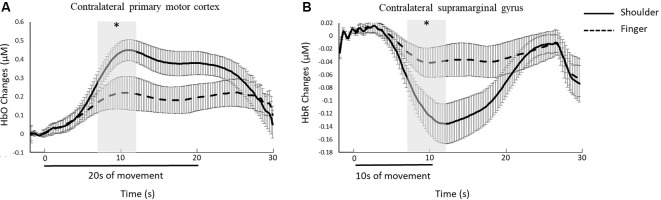
Group average results for **(A)** HbO changes in the left primary motor cortex (channel 28) during shoulder-20 s and finger-20 s conditions and **(B)** HbR changes in left supramarginal gyrus (channel 37) during shoulder-10 s and finger-10 s conditions. Shaded bars indicate the interval chosen to obtain the mean responses. Asterisks indicate significant differences between tasks. Error bars represent the standard error across participants.

Significant differences in task-related hemodynamic responses as indicated by HbO and HbR between different motor tasks were found; however, no significant difference between different trial lengths was found. For HbO, significantly greater activation in the contralateral primary motor cortex (channel 28) was found during the shoulder-20 s condition compared to the finger-20 s condition (Figure 5A). No significant differences in HbO between shoulder-10 s and finger-10 s conditions, between shoulder-10 s and shoulder-20 s, or finger-10 s and finger-20 s conditions were found. Also, a significantly greater activation as shown by a greater decrease in HbR in the contralateral supramarginal gyrus (channel 37) was found in the shoulder-10 s condition compared to the finger-10 s condition (Figure 5B). No significant differences in HbR between shoulder-20 s and finger-20 s conditions, between shoulder-10 s and shoulder-20 s, or finger-10 s and finger-20 s conditions were found.

### Comparisons of Task-Related Hemodynamic Responses in Primary Motor Cortex Between Hemispheres

Significant differences were found in the task-related hemodynamic responses in primary motor cortex between hemispheres. For HbO, a significantly greater increase in HbO was found in contralateral compared to ipsilateral primary motor cortex in shoulder-10 s, shoulder-20 s, finger-10 s, and finger-20 s conditions (for channel 26 vs. 32: *p* < 0.001, *p* < 0.001, *p* = 0.001, *p* < 0.001; for channel 28 vs. 33: *p* = 0.003, *p* < 0.001, *p* < 0.001, *p* = 0.12). For HbR, a significantly greater decrease was found in contralateral compared to ipsilateral primary motor cortex in shoulder-10 s, shoulder-20 s, finger-10 s, and finger-20 s conditions (for channel 26 vs. 32: *p* = 0.005, *p* < 0.001, *p* = 0.012, < 0.001; for channel 28 vs. 33: *p* = 0.26, *p* = 0.76, *p* = 0.016, *p* = 0.72).

### Behavioral Data

For the conditions with trial length of 10 s, participants performed an average of 5.50 repetitions (SD = 0.24) for shoulder-10 s condition and 5.60 repetitions (SD = 0.17) for finger-10 s conditions. For the conditions with trial length of 20 s, participants performed an average of 10.45 repetitions (SD = 0.30) for shoulder-20 s condition and 10.55 repetitions (SD = 0.16) for finger-20 s conditions. No significant differences in the number of repetitions were found between conditions with the same trial length (for 10 s conditions, *p* = 0.07; for 20 s conditions, *p* = 0.18).

## Discussion

Consistent with our first hypothesis, the study showed that performing upper extremity motor tasks produced a generalized bilateral cortical activation in motor- and sensory-related cortical areas with greater activation in the contralateral hemisphere in comparison with the ipsilateral side in healthy adults. Specifically, greater neural activation in the more medial part of the contralateral primary motor cortex was found during the shoulder condition compared to the finger condition. These findings are relevant for understanding recovery mechanisms and predicting a motor recovery in individuals with neurological disorders such as stroke. Partially contrary to our second hypothesis, different trial lengths influence cortical activation patterns but not the level of activation during the same motor tasks. More active areas as shown by significant increases in HbO were found during finger movements in longer trials (i.e., 20 s) compared to shorter trials (i.e., 10 s), whereas more active areas as indicated by significant decreases in HbR were found during shoulder movements in shorter trials compared to longer trials.

From a neuroanatomical standpoint, it is not surprising that bilateral cortical activation was demonstrated during the abduction of the shoulder due to bilateral innervations of proximal muscles (Colebatch et al., 1990; Bawa et al., 2004; Schwerin et al., 2008). Although unilateral distal movements such as the fingers have been understood to be controlled primarily by the contralateral hemisphere, previous literature shows the ipsilateral primary motor cortex is also involved with finger movement (Babiloni et al., 1999; Kinoshita et al., 2000; Muellbacher et al., 2000; Luft et al., 2002). Such involvement from ipsilateral cortical hemisphere has been proposed to be associated with interhemispheric inhibition, keeping an efferent copy of the ipsilateral limb state to coordinate bimanual movements, postural control of proximal musculature, and specific roles of each hemisphere during planning and execution of voluntary movements (Bundy and Leuthardt, 2019). As our movement tasks were performed only with the ipsilateral upper limb in a seated position with back supported, which did not involve bilateral coordination and required similar demands for posture control between conditions, it is unlikely that the maintaining an efferent copy for bilateral coordination and the involvement of proximal musculature could account for the observed ipsilateral cortical activation during the finger extension task. Therefore, the cortical activity in the ipsilateral hemisphere during shoulder abduction and finger extension tasks may result from possible inhibition in the ipsilateral hemisphere (Duque et al., 2007) and/or active contributions from the ipsilateral hemisphere (Schaffer and Sainburg, 2017).

Our results demonstrated a greater neural activation as shown by higher HbO during shoulder abduction compared with finger extension in the more medial part of the contralateral primary motor cortex (Brodmann area 4), which is consistent with the homunculus map of a human brain. We also found a greater neural activation during shoulder abduction as shown by a greater decrease in HbR in the medial part of the supramarginal gyrus (Brodmann area 40, part of the somatosensory association cortex), which has roles in combining and integrating inputs from several brain regions (Whitlock, 2017). The results are consistent with the findings in a previous study by Yeo et al. (2013), which only recorded the contralateral hemisphere and found a greater increase in HbO in the primary sensorimotor cortex during movements of the shoulder compared with hand movements. However, they also found greater cortical activation in the premotor cortex and prefrontal cortex during movements of the shoulder compared with hand movements. The motor tasks in Yeo’s study were flexion-extension movements of the shoulder or hand in a supine position whereas the tasks in our study were abduction of the shoulder or extension of the fingers in a seated position. Head and body positioning have been shown to influence brain oxygenation captured by fNIRS (Fuchs et al., 2000). The different demands for motor tasks and body positions may explain the differences between these studies. Interestingly, no significant differences in the level of cortical activation were found in other sensorimotor areas between shoulder abduction and finger extension tasks. As cortical activation is known to be dependent on muscle force (Derosiere and Perrey, 2012), we anticipate observing a generally greater level of cortical activation during shoulder abduction compared to finger extension. We suspect that the level of cortical activation may not only depend on muscular effort but also the area of motor and sensory representation in the brain.

Typically, a hemodynamic response function during neurovascular coupling recorded by the fNIRS instrument is characterized by an increase in HbO with a concurrent small decrease in HbR (Phillips et al., 2016). The majority of literature in task-evoked cortical activation using fNIRS has focused on the explanation of changes in HbO but has under-reported the results of HbR. However, each hemoglobin species is representative of hemodynamic responses and has its challenges as a surrogate measure of the neurovascular coupling to brain activation. HbO is more likely to be contaminated with a systemic physiological artifact such as heart rate and blood pressure, while HbR is less consistent due to a larger intersubject variability (Sato et al., 2005). In this study, we observed that different hemoglobin species might have different responses to motor paradigms including trial lengths and motor tasks. These findings are partially consistent with our second hypothesis, which hypothesize no difference in activation patterns between shorter and longer trial lengths. More channels are activating as shown by a significant increase in HbO during finger-20 s condition in comparison with finger-10 s condition. In contrast, more channels activating as shown by a significant decrease in HbR during shoulder-10 s in comparison with the shoulder-20 s condition. HbO has a higher signal to noise ratio than HbR (Strangman et al., 2002) and more sensitive to regional flow changes (Hoshi, 2003, 2016). As a consequence, it is likely that larger amplitude changes in HbO during longer trials (20 s of finger extension) with more repetitions, result in greater sensitivity in detecting activation in comparison with shorter trials (10 s of finger extension). Furthermore, different motor tasks may evoke distinct systemic physiological changes (e.g., heart rate, respiration rate, or blood pressure, etc.) affecting oxygenation and blood volume in the brain, which may have a differential influence on the signal of HbO and HbR (Tachtsidis and Scholkmann, 2016). Although the short separation channels were used to minimize the effects of systemic physiological changes on the fNIRS signal, it is likely that the physiological noise was not entirely eliminated (Gagnon et al., 2014). Therefore, each hemoglobin species may have its advantages to detect activation in different motor paradigms. In future research studies, we suggested to analyze and report all the available hemoglobin data in fNIRS to better understand the task-evoked cortical activation patterns. A trial length of 10 s of movement detected several common brain regions areas (e.g., contralateral premotor, supplementary motor cortex, primary motor cortex) that were also found in a trial length of 20 s. Hence, we suggest that trial length of 10 s is sufficient to gather information regarding regional brain activation differences especially for clinical application in participants who fatigue easily.

There are several limitations to this study. First, the number of participants was small, and we did not have individual MRI data to confirm the spatial registration of each channel on the brain. Although we used the “fOLD” toolbox to guide the design of the probe to cover the cortical regions of interest, the precise cortical area that each fNIRS channel measured was not confirmed. Further accuracy of channel locations could have been attained through using 3D digitizations of channel locations and co-registering these to template atlas brains or individual MRI. Despite this limitation, our study still showed distinct patterns of cortical activation generated by movements of shoulder and fingers. Larger sample size may be needed for future studies in neurological populations given the variability in the human brain with neurological conditions. Second, the findings are not able to generalize to left-handed participants and motor tasks performed on the non-dominant side. In this study, only right-handed participants were recruited, and motor tasks were only performed on the dominant right side. It is well known that each hemisphere is specialized in the control of upper limb movements and that handedness affects motor control of upper limbs based on the dynamic-dominance hypothesis (Schaffer and Sainburg, 2017). Although we decided to focus on right-handed participants and the dominant side to make the study well-focused, future experiments on left-handed participants should be conducted to confirm the effects of handedness and asymmetry of the cerebral hemisphere on task-related cortical activation. Third, only two trial lengths (i.e., 10 s and 20 s) were examined, and motor tasks were performed at 0.5 Hz. Future fNIRS studies should examine the minimum trial length (e.g., 3, 5, 7 s, etc.) and movement speed required to capture cortical activation patterns especially in people experiencing fatigue easily (Khan et al., 2020). Lastly, although none of our participants were professional musicians or athletes, we did not collect information on specific musical or athletic experiences. While these previous experiences may influence the amount of representative cortex associated with the shoulder or finger, the movements performed within this study were simple movements that were easily performed by all participants. Specific participant exclusion based on previous experience would be required if more complex or fine control tasks were performed as these movements have shown differential brain activations between musicians and non-musicians (Pau et al., 2013).

## Conclusion

Our study showed the cortical activation patterns associated with shoulder abduction and finger extension captured by fNIRS in healthy adults. As the ability to perform such movements has been shown to predict upper extremity functional recovery following stroke, the findings are relevant for the predictive evaluation of upper extremity motor recovery in individuals with stroke or other neurological disorders. Diagnosis and prediction of motor recovery in this population are beneficial for clinicians to develop optimized rehabilitation strategies and efficient usage of healthcare resources. By utilizing all fNIRS channels covering bilateral sensorimotor and frontal cortices, we have established the regions of activation for a given task, which would serve a guide for the selection of regions of interest for future studies. We further demonstrated that the cortical activation can be detected by different surrogate measurements (i.e., HbO and HbR) of neurovascular coupling to brain activation and the detection of activation was affected by motor tasks (shoulder vs. finger movements) and trial lengths (10 s vs. 20 s). Based on our results, we strongly recommend reporting both HbO and HbR hemoglobin species for all fNIRS studies. fNIRS is less time- and resources- consuming, as well as allows for a faster evaluation of brain function and more freedom of body movements during measurement compared to traditional neuroimaging methods. The new insight revealed from the study would be beneficial for clinical application and implementation of the fNIRS in the disease population.

## Data Availability Statement

The raw data supporting the conclusions of this article will be made available by the authors, without undue reservation.

## Ethics Statement

The studies involving human participants were reviewed and approved by the University of British Columbia Behavioural Research Ethics Board. The patients/participants provided their written informed consent to participate in this study.

## Author Contributions

C-LY devised the project under the supervision of JE. C-LY conducted data collection, data analysis, and drafted the manuscript. All authors provided critical feedback and helped shape the research, analysis, and manuscript.

## Conflict of Interest

The authors declare that the research was conducted in the absence of any commercial or financial relationships that could be construed as a potential conflict of interest.
